# Specific contribution of mannose-binding lectin murine isoforms to brain ischemia/reperfusion injury

**DOI:** 10.1038/s41423-019-0225-1

**Published:** 2019-04-09

**Authors:** Laura Neglia, Marco Oggioni, Domenico Mercurio, Maria-Grazia De Simoni, Stefano Fumagalli

**Affiliations:** Istituto di Ricerche Farmacologiche Mario Negri IRCCS, Department of Neuroscience, Milan, Italy

**Keywords:** Complement cascade, Neuroimmunology

## Abstract

Mannose-binding lectin (MBL), an initiator of the lectin pathway (LP) of complement activation, is detrimental in ischemic stroke, as shown in clinical studies and rodent models. Whereas humans have one functional MBL protein, rodents have two isoforms, MBL-A and MBL-C, whose functions relative to that of human MBL are unknown. To permit the clinical translation of preclinical data, we aimed to define the specific contributions of MBL-A and MBL-C to brain ischemia. We subjected mice with double (MBL^−/−^) or single (MBL-A^−/−^ or MBL-C^−/−^) MBL isoform depletion to transient middle cerebral artery occlusion (tMCAo). MBL^−/−^ mice had fewer neurological deficits and smaller ischemic lesions than WT mice. MBL-A^−/−^ mice had smaller lesions than WT mice and exhibited no significant behavioral defects, whereas MBL-C^−/−^ mice did not differ from WT mice. The induction of *Mbl1* and *Mbl2* (the MBL-A and MBL-C genes) expression 48 h after tMCAo was similar across genotypes. The time course of *Mbl1* and *Mbl2* expression in WT ischemic mice showed that *Mbl1* activation occurred earlier (24 h) than *Mbl2* activation (48 h). The plasma levels of MBL-A and MBL-C in MBL-C^−/−^ and MBL-A^−/−^ mice, respectively, were similar to those in WT mice both at baseline and at 48 h after tMCAo. At 48 h, MBL-A^−/−^ ischemic mice showed higher MBL-C levels in the brain than WT mice. WT and MBL-C^−/−^ ischemic mice had higher LP activity in plasma and, accordingly, higher levels of C3 deposition in the brain than MBL-A^−/−^ and MBL^−/−^ mice. In conclusion, mice with depletion of both MBL isoforms exhibited strong protection from ischemia/reperfusion injury. MBL-A was the main contributor to injury, likely owing to its earlier activation after ischemia and more efficient activation of the complement system than MBL-C.

## Introduction

Mannose-binding lectin (MBL) is a soluble receptor in the innate immune system that binds to pathogen- or damage-associated molecular patterns, inducing the activation of the complement system through a proteolytic cascade. In particular, MBL is one of the initiators of the lectin pathway (LP) of complement activation, which has been reported to play a key pathogenic role in ischemia/reperfusion injury in murine models and in stroke patients. MBL-depleted mice show smaller ischemic lesions and fewer deficits after ischemia than their wild-type counterparts,^1,2^ and these data are paralleled in humans.^3^ Genetic variants cause MBL deficiency in ~ 20% of the general population,^4–7^ and stroke patients with MBL deficiency have smaller infarctions and better outcomes than those without,^1,8^ supporting the hypothesis of a key role of MBL in human stroke pathophysiology.

MBL is a circulating protein that is mainly synthesized in the liver. The working hypothesis for MBL’s detrimental action in the brain considers its deposition in the damaged endothelium as the event precipitating the pathological consequences.^2,3^ Although most mechanisms of action remain unclear, MBL is currently considered a hub in vascular injury, being activated rapidly in response to early danger signals and controlling/coordinating multiple pathogenic cascades.^3^ New evidence indicates that MBL, in addition to activating the LP, activates prothrombotic cascades via its associated serine proteases^9–11^ and directly activates an inflammatory phenotype in platelets.^12^ Thus, MBL offers a promising therapeutic target for brain ischemia—it is systemically accessible and controls many mechanisms of injury. Our group previously demonstrated that pharmacological targeting of MBL by intravenous injection of an anti-MBL antibody or Polyman2, a mannosylated molecule acting as an MBL inhibitor,^2,13^ improves neurological deficits and reduces the severity of cerebral lesions in ischemic rats and mice. These treatments were effective when administered up to 18–24 h after injury, indicating a wide therapeutic window^2^ and possibly supporting the implication of MBL in different temporal events. Depletion or pharmacological targeting of MBL was also neuroprotective in a model of traumatic brain injury, a condition sharing some vascular consequences with ischemia.^14,15^

Whereas rodents have two functional MBL isoforms (MBL-A and MBL-C),^16^ humans have only one; thus, a clear characterization of the rodent models is needed to permit the clinical translation of promising preclinical data. Data have been reported for double MBL isoform knockout mice^1,2,14^ and for molecules with preferential but nonexclusive action on a single MBL isoform.^2,15^

To define the specific contributions of MBL-A and MBL-C to brain ischemia, we developed single MBL isoform-depleted mice (MBL-A^−/−^ or MBL-C^−/−^ mice) and investigated their susceptibility to cerebral ischemic injury. At 48 h after the induction of focal transient ischemia, we analyzed behavioral deficits, histological parameters (ischemic volume and neuronal viability), hepatic *Mbl1* and *Mbl2* gene expression (coding for MBL-A and MBL-C, respectively), MBL isoform plasma levels and complement system activation systemically and in the brain.

## Results

**Fig. 1:**
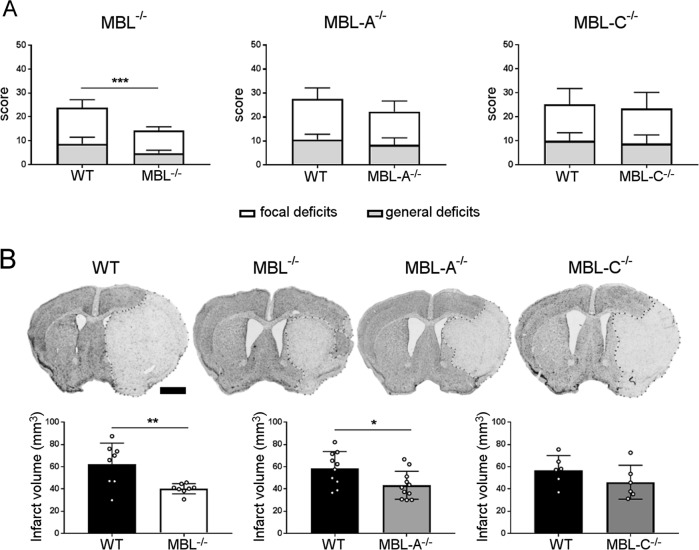
Neuroscore and ischemic volume after 48 h of reperfusion in MBL^−/−^, MBL-A^−/−^, and MBL-C^−/−^ mice. **a** Only MBL^−/−^ mice had fewer behavioral deficits than WT mice when rated by the composite neuroscore (the sum of focal and general deficits). The data are shown as bars + SDs (*n* = 7 for MBL^−/−^, *n* = 10 for MBL-A^−/−^, and *n* = 6 for MBL-C^−/−^); Mann–Whitney test, ***p < 0.001. **b** Representative images of cresyl violet-stained sections, scale bar 1 mm. MBL^−/−^ and MBL-A^−/−^ mice had a smaller ischemic volume than WT mice. The data are shown as bars with individual values ± SDs (*n* = 7 for MBL^−/−^, *n* = 10 for MBL-A^−/−^, and *n* = 6 for MBL-C^−/−^); *t* test, **p < 0.01, *p < 0.05

**Fig. 2:**
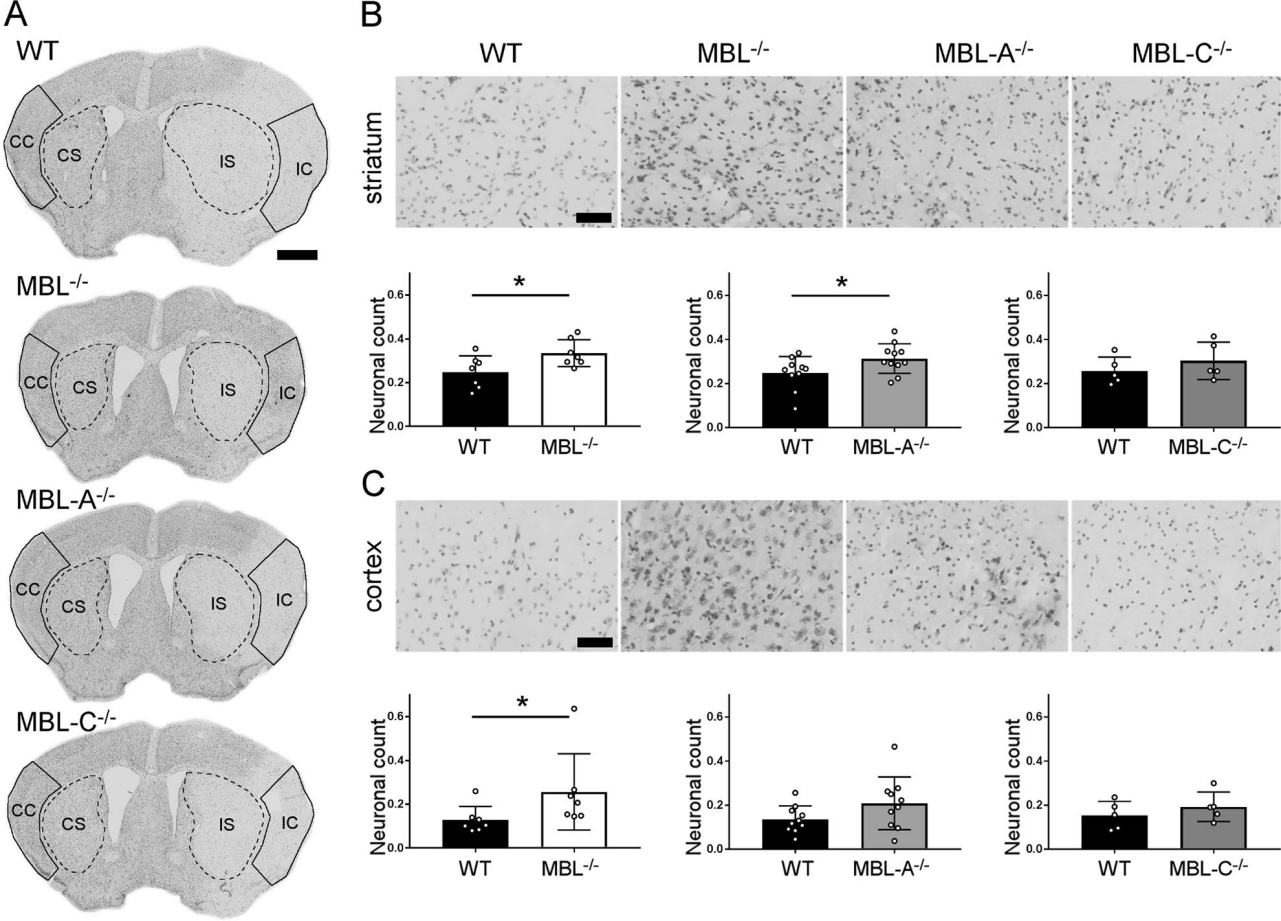
Neuronal count after 48 h of reperfusion in MBL^−/−^, MBL-A^−/−^, and MBL-C^−/−^ mice. **a** Positioning of the cortical (outlines) and striatal (dotted outlines) regions of interest for calculating neuronal cell viability (CC: contralateral cortex; CS: contralateral striatum; IC: ipsilateral cortex; IS: ipsilateral striatum). The regions of interest were designed to include only the lesion area (pale cresyl violet staining) throughout the experimental groups. **b**, **c** Representative high-magnification images of cresyl violet-stained sections (scale bar, 50 µm) and neuronal counts showing that MBL^−/−^ mice had more preserved neurons in the striatum and cortex and MBL-A^−/−^ mice had more preserved neurons in the striatum than WT mice. The data are shown as bars with individual values ± SDs (*n* = 7 for MBL^−/−^, *n* = 10 for MBL-A^−/−^, and *n* = 5 for MBL-C^−/−^); *t* test, *p < 0.05. Compared with WT mice, MBL-C^−/−^ mice showed no protection from ischemic injury

The susceptibility of MBL^−/−^, MBL-A^−/−^, and MBL-C^−/−^ mice to brain ischemia was evaluated 48 h after ischemic onset, when the ischemic lesion was fully developed in the transient middle cerebral artery occlusion (tMCAo) model.^2^ Compared with WT mice, MBL^−/−^ ischemic mice had a 40% reduction (*p* < 0.001), whereas MBL-A^−/−^ and MBL-C^−/−^ mice had a nonsignificant reduction (19% and 7%, respectively) in composite neurological deficits (Fig. 1a). MBL^−/−^ and MBL-A^−/−^ mice had smaller lesions than WT mice (35% and 26%, *p* < 0.01 and *p* < 0.05, respectively), whereas MBL-C^−/−^ mice had a nonsignificant reduction in lesion size (19%, Fig. 1b). Similarly, MBL^−/−^ mice had higher neuronal viability in the striatum and cortex (35% and 99%, respectively, *p* < 0.05) and MBL-A^−/−^ but not MBL-C^−/−^ mice had higher neuronal viability in the striatum than WT mice (27%, *p* < 0.05) (Fig. 2b, c).

**Fig. 3:**
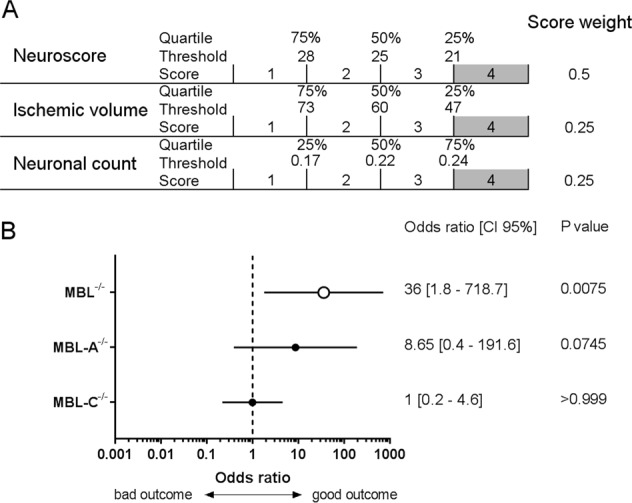
Association of double or single isoform MBL deficiency with ischemic outcome at 48 h. **a** The “healthy score” was obtained by rating mice from 4 (good outcome) to 1 (poor outcome) based on quartiles of neuroscore, ischemic volume and neuronal count (summed over the cortex and striatum quantifications). The final score was the sum of the weighted scores of the three parameters, e.g., the neuroscore accounted for 50% and the ischemic volume and neuronal count each accounted for 25% of the final score. **b** Association (odds ratio; 95% confidence interval) between the genotype and “healthy score”. The odds ratio was calculated by a Chi-square test using the Woolf logit interval for computing the 95% confidence interval (CI 95%), stratifying mice in terms of good outcome (defined as a score = 4) vs. bad outcome (score < 4). Only MBL^−/−^ mice were significantly associated with a good outcome

To obtain comprehensive data on the three outcome measures of ischemia (neuroscore, ischemic volume and neuronal count, summarized in Table 1), we developed a “healthy score” rating ischemic mice from 4 (good outcome) to 1 (bad outcome, Fig. 3a). MBL^−/−^ mice were clearly protected, with double MBL isoform knockout showing a positive association with a good outcome (*p* = 0.0075); the MBL-A^−/−^ genotype showed a weaker, nonsignificant association with a good outcome (*p* = 0.0745), whereas MBL-C^−/−^ mice were not protected (*p* > 0.999, Fig. 3b).

**Table 1:** Summary of the outcome measures in the MBL^−/−^, MBL-A^−/−^, or MBL-C^−/−^ ischemic mice. Neuronal count represent the global value relative to striatum and cortex

Strain		% than WT	*P* value
MBL^−/−^			
	Neuroscore	−40%	0.0004***
	Ischemic volume	−35%	0.0070**
	Neuronal count	+48%	0.0445*
MBL-A^−/−^			
	Neuroscore	−19%	0.1674
	Ischemic volume	−26%	0.0220*
	Neuronal count	+42%	0.0389*
MBL-C^−/−^			
	Neuroscore	−7%	0.7489
	Ischemic volume	−19%	0.2157
	Neuronal count	+23%	0.2026

Statistical analysis done as described in methods, *p < 0.05, **p < 0.01, ***p < 0.001 vs. WT

**Fig. 4:**
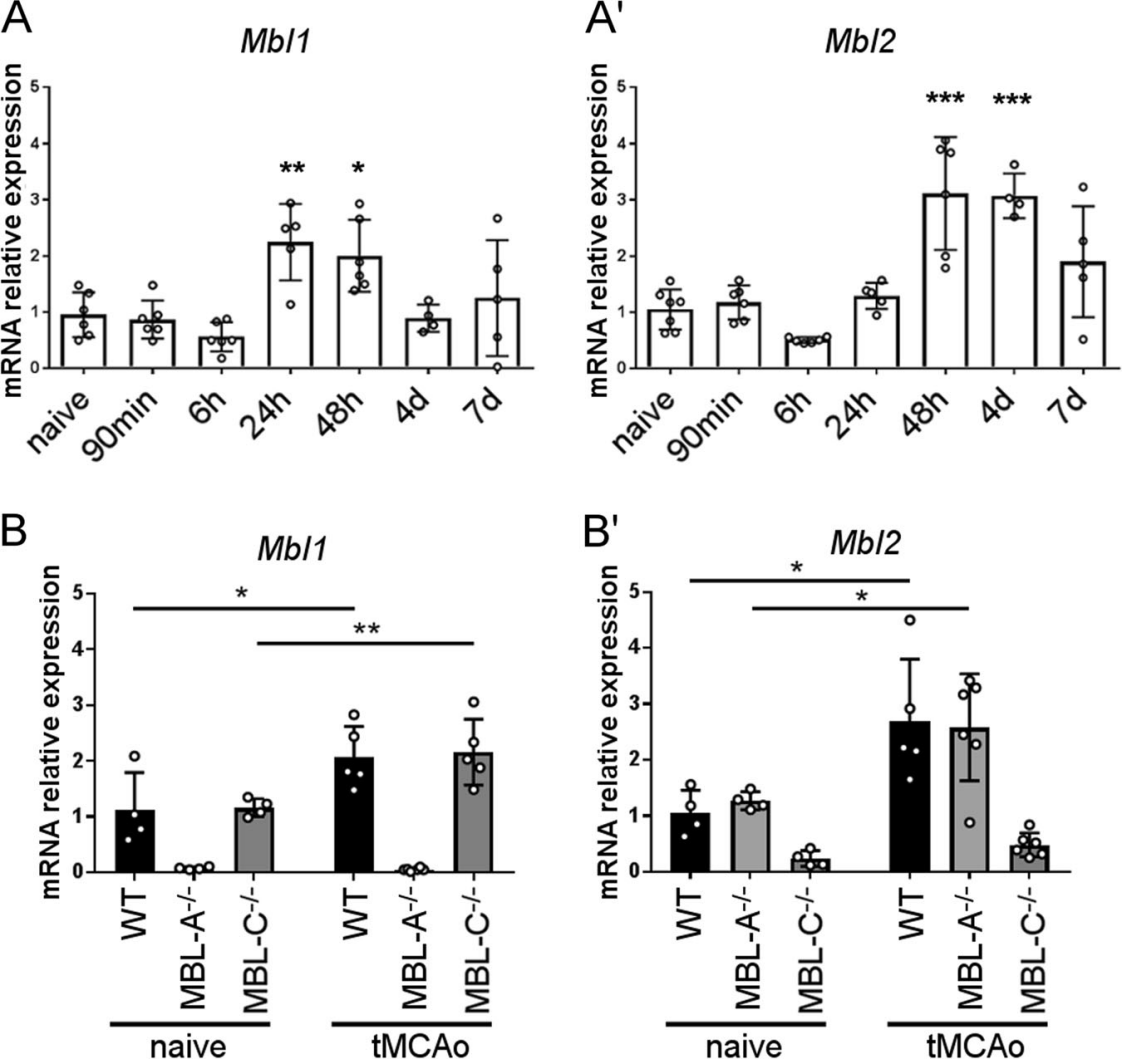
Hepatic gene expression of *Mbl1* and *Mbl2* after 48 h of reperfusion in MBL^−/−^, MBL-A^−/−^, and MBL-C^−/−^ mice. **a**, The time course of *Mbl1* (**a**) and *Mbl2* (**a’**) hepatic expression in WT mice showed overexpression of *Mbl1* at 24 h and 48 h and overexpression of *Mbl2* at 48 h and 4 d after tMCAo. The data are shown as bars with individual values ± SDs (*n* = 5–6); one-way ANOVA followed by Dunnett’s post hoc test, *p < 0.05, **p < 0.01, ***p < 0.001 vs. naive. **b** At 48 h after tMCAo, *Mbl1* hepatic expression in MBL-C^−/−^ mice was induced to the same extent as that in WT mice (**b**). The data are shown as bars with individual values ± SDs (*n* = 4–6); two-way ANOVA followed by Sidak’s post hoc test, *p < 0.05, **p < 0.01. At 48 h after tMCAo, *Mbl2* hepatic expression in MBL-A^−/−^ mice was induced to the same extent as that in WT mice (**b'**). The data are shown as bars with individual values ± SDs (*n* = 4–6), Welch-corrected ANOVA for unequal variances, *p < 0.05

**Fig. 5:**
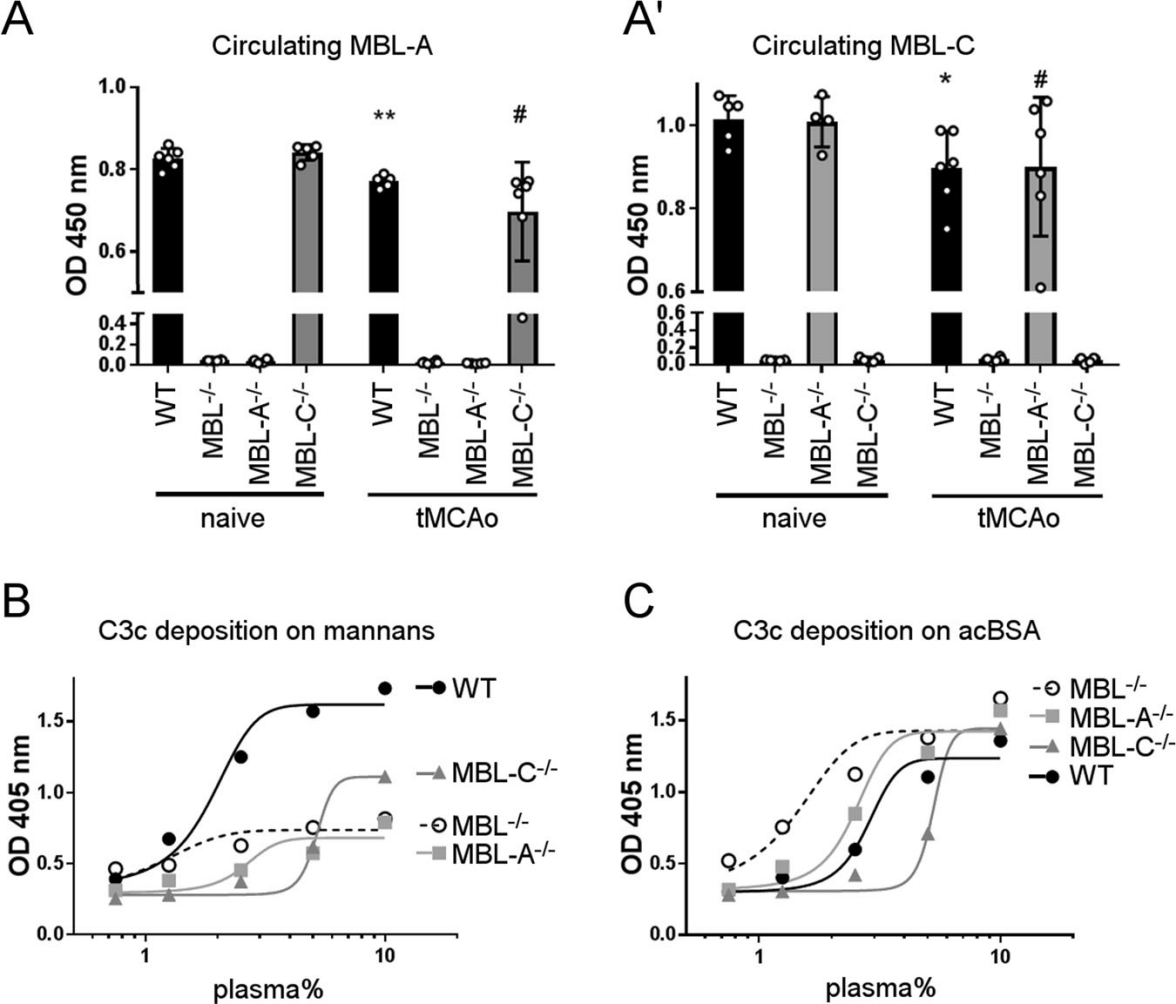
Plasma levels of MBL-A and MBL-C and systemic activation of the complement system after 48 h of reperfusion in MBL^−/−^, MBL-A^−/−^, and MBL-C^−/−^ mice. **a** At 48 h after tMCAo, MBL-A was consumed to the same extent in MBL-C^−/−^ mice as in WT mice (**a**). The data are shown as bars with individual values ± SDs (*n* = 5–6); Welch-corrected ANOVA for unequal variances, *p < 0.05 vs. naive WT mice, ^#^*p* < 0.05 vs. naive MBL-C^−/−^ mice. At 48 h after tMCAo, MBL-C was consumed to the same extent in MBL-A^−/−^ mice as in WT mice (**a'**). The data are shown as bars with individual values ± SDs (*n* = 5–6); two-way ANOVA followed by Sidak’s post hoc test, *p < 0.05 vs. naive WT mice, ^#^*p* < 0.05 vs. naive MBL-C^−/−^ mice. **b** The in vitro assay for LP activation on mannans showed no activation in plasma from MBL^−/−^ and MBL-A^−/−^ mice but some activation in plasma from MBL-C^−/−^ mice. **c** The in vitro assay for LP activation on acBSA showed similar activation in plasma from mice of each genotype. The data in **b** and **c** refer to pools of plasma from 5–6 mice per group, and plasma concentrations are reported on a logarithmic scale

We next analyzed the time course of *Mbl1* and *Mbl2* gene expression (coding for MBL-A and MBL-C, respectively) in the liver, the main site of MBL production,^16^ in WT ischemic mice. Ischemia induced *Mbl1* overexpression at 24 h (2.25 ± 0.68 (times more than that in naive mice ± SD)) and 48 h (2.00 ± 0.64); thus, the increase in *Mbl1* expression preceded that of *Mbl2* expression (observed at 48 h, 3.12 ± 1.00, and 4 d, 3.08 ± 0.40; Fig. 4a). In a second experimental set, we analyzed *Mbl1* and *Mbl2* hepatic gene expression at 48 h in single MBL isoform-deficient mice. MBL-C^−/−^ and WT mice had similar levels of *Mbl1* overexpression (2.16 ± 0.59 vs. 2.07 ± 0.55), and MBL-A^−/−^ and WT mice had similar levels of *Mbl2* overexpression (2.58 ± 0.95 vs. 2.69 ± 1.11, Fig. 4b). This overexpression is a response to MBL-A and MBL-C protein usage that causes their consumption and consequently reduces their circulating protein level. In line with this finding, WT ischemic mice had lower circulating levels of MBL-A (0.77 ± 0.01, OD ± SD) and MBL-C (0.90 ± 0.09) than naive mice (MBL-A: 0.83 ± 0.02 and MBL-C: 1.02 ± 0.56). MBL-C^−/−^ ischemic mice had lower MBL-A levels than naive mice (0.70 ± 0.12 vs. 0.84 ± 0.02). MBL-A^−/−^ ischemic mice had lower MBL-C levels than naive mice (0.901 ± 0.17 vs. 1.01 ± 0.06, Fig. 5a). No differences in the consumption of the remaining MBL protein isoform were found in single isoform knockout mice compared with that in WT mice.

We tested the activation of the LP in mouse plasma (pools from mice belonging to the same experimental group) using an in vitro assay^17^ with mannan-coated plates, which measures LP activation through MBL.^18^ There was no LP activation in MBL^−/−^ and MBL-A^−/−^ ischemic mice, whereas the LP remained active in MBL-C^−/−^ mice, though to a lesser extent than in WT mice (Fig. 5b). The in vitro assay using acBSA-coated plates, which measures LP activation through ficolins,^18^ did not show any difference among the genotypes (Fig. 5c).

**Fig. 6:**
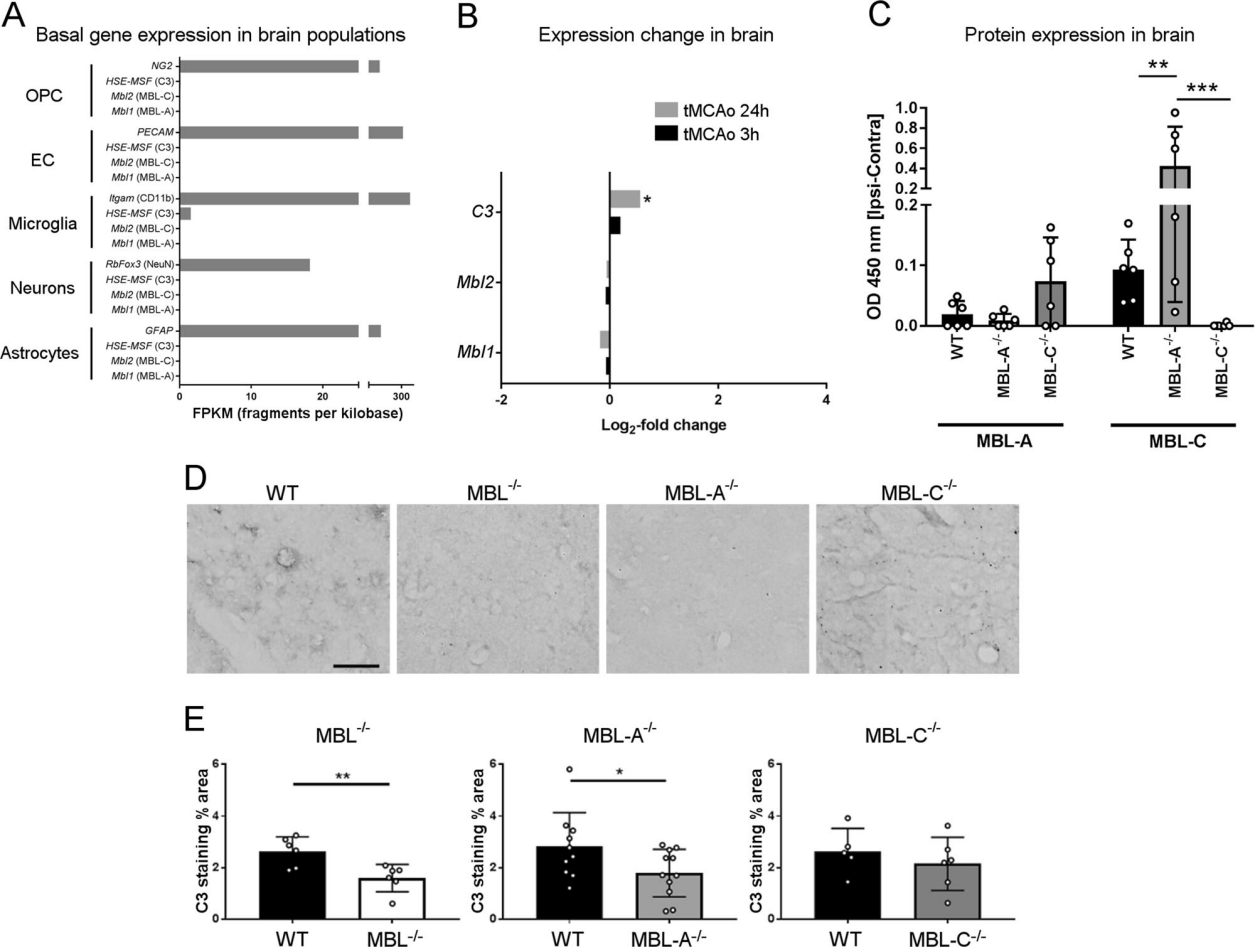
Gene and protein expression levels of MBL isoforms and complement system activation in the brain after ischemia/reperfusion in WT, MBL^−/−^, MBL-A^−/−^, and MBL-C^−/−^ mice. **a** Basal gene expression (fragments per kilobase million, FKPM) of *Mbl1*, *Mbl2*, and *HSE-MSF* (C3) in *Mus musculus* brain cell populations. Oligodendrocyte precursors (OPCs, expressing *NG2*), endothelial cells (ECs, expressing *PECAM*), microglia (expressing *Itgam*), neurons (expressing *RbFox3*), and astrocytes (expressing *GFAP*) do not express *Mbl1* or *Mbl2*. Microglia are the only brain cell population expressing *HSE-MSF* (C3). Data were obtained from single-cell RNA-seq databases as described in the Materials and methods section. **b** Expression changes (microarray analysis) of *Mbl1*, *Mbl2*, and *HSE-MSF* (C3) in the brain following brain ischemia/reperfusion in WT mice. *Mbl1* and *Mbl2* gene expression was not induced at 3 h or 24 h after tMCAo. C3 expression was significantly upregulated at 24 h after tMCAo. The data are expressed as the log_2_ fold change compared with untreated mice, with a Benjamini–Hochberg multiple comparisons test for *P* value adjustment, *p < 0.05 vs. untreated mice (*n* = 4 for each experimental group). Data were obtained from microarray databases as described in the Materials and methods section. **c** MBL-A and MBL-C protein presence in the brain cortices of WT, MBL-A^−/−^, and MBL-C^−/−^ mice 48 h after tMCAo. MBL-A was present in WT and MBL-C^−/−^ mice to a similar extent. MBL-C was present to a higher extent in MBL-A^−/−^ mice than in WT mice. The data are expressed as the differences between the ipsilateral and contralateral optical densities (OD 450 nm) and are shown as bars with individual values ± SDs (*n* = 6); two-way ANOVA followed by Sidak’s post hoc test, **p < 0.01 and ***p < 0.001. **d** Representative high-magnification images of C3 immunolabeling at 48 h after tMCAo, scale bar 50 µm. **e** MBL^−/−^ and MBL-A^−/−^ but not MBL-C^−/−^ mice had less C3 deposition in the cortex than WT mice. The data are shown as bars with individual values ± SDs (*n* = 6–10); *t* test, **p < 0.01, *p < 0.05

We then assessed the presence of the LP in the brain. *Mbl1* and *Mbl2* are not expressed by brain cells in mice under basal conditions, as indicated by transcriptomic data obtained using single-cell RNA-seq (Fig. 6a). The gene coding for complement C3 (*HSE-MSF*) is expressed by microglia, consistent with the observation that the homeostatic function of microglia involves synaptic pruning through opsonins such as C3.^19^ Ischemia/reperfusion injury did not induce *Mbl1* or *Mbl2* expression in the brain. Instead, C3 was upregulated after 24 h of reperfusion, as shown by analysis of the microarray databases (Fig. 6b). ELISA on brain cortical homogenates revealed the presence of both isoforms in the brain of ischemic mice after 48 h of reperfusion, with MBL-C significantly more abundant in MBL-A^−/−^ mice than in WT mice (0.427 ± 0.387 vs. 0.093 ± 0.049 (optical density ± SD), Fig. 6c). Consistent with the data on the in vitro activation of the LP through MBL and those on the presence of MBL in the brain, we found that C3 deposition in the brain cortex, a hallmark of systemic complement activation,^20^ was reduced in MBL^−/−^ (39%, *p* < 0.01) and MBL-A^−/−^ (37%, *p* < 0.05) but not in MBL-C^−/−^ (a nonsignificant 18% reduction) ischemic mice compared with that in WT mice (Fig. 6d, e).

## Discussion

We analyzed the consequences of double and single MBL isoform depletion in a mouse model of focal brain ischemia with reperfusion. In line with previous findings,^1,2^ mice depleted of both isoforms were protected from ischemic injury, showing fewer behavioral deficits, smaller ischemic volume and a larger number of viable neurons than WT ischemic mice. Depletion of a single MBL isoform induced low-level (MBL-A^−/−^) or negligible (MBL-C^−/−^) neuroprotection from ischemic injury compared with that achieved when both isoforms were depleted. MBL-A depletion reduced neuronal death and ischemic volume but caused only a small, nonsignificant reduction in behavioral deficits. MBL-C depletion partially reduced ischemic volume and neuronal death, although none of these effects were significant. We therefore propose MBL-A as the main isoform participating in the development and evolution of ischemic brain injury, but pathophysiological roles for both MBL isoforms should be considered. In line with a role for both isoforms, only MBL^−/−^ mice exhibited protection when the three outcomes (neurological deficits, ischemic volume, and neuronal count) were combined into a comprehensive “healthy score”. With respect to clinical translatability, functional recovery in preclinical models should be considered a priority. Thus, in the “healthy score”, the highest weighting was attributed to the neuroscore. Although a possible limitation of the score is the interindependence of the variables, it provides an objective estimate of the functional role of the two isoforms.

According to a previous characterization, murine MBL-A and MBL-C have similar circulating concentrations (from 5 to 40 μg/mL in laboratory mouse strains) and oligomerization profiles.^16^ However, MBL-C was reported to be approximately one-fifth less active than MBL-A in inducing complement activation in vitro.^16^ Although this difference may not be mirrored in vivo, our data showing a decrease in ischemic volume and increase in neuronal viability in MBL-A^−/−^ but not MBL-C^−/−^ mice support the previous observations and suggest that MBL-A is the main activator of the complement system after brain ischemia. Here, a functional in vitro LP activation assay on plasma samples from ischemic mice showed residual LP activity in MBL-C^−/−^ but not MBL-A^−/−^ mice. Moreover, the deposition of C3 in the brain, an index of complement activation, was lower in MBL-A^−/−^ but not MBL-C^−/−^ ischemic mice than in WT ischemic mice. Notably, MBL-A^−/−^ mice had a significantly higher MBL-C content than WT mice 48 h after tMCAo, likely owing to a lack of competition for endothelial ligands in the absence of MBL-A. Similarly, MBL-C^−/−^ mice had increased levels of MBL-A in the brain, although this increase was nonsignificant. The lack of obvious MBL-A presence at the analyzed time point (48 h) agreed with the earlier appearance of MBL-A in the brain.^2^ Although MBL-A^−/−^ mice exhibited increased MBL-C levels in the brain, they showed lower brain complement activation and brain damage than WT mice, lending further support to the hypothesis that MBL-C is a less efficient driver of LP activation and has a smaller impact than MBL-A on brain ischemia/reperfusion pathophysiology. Current evidence indicates that MBL can activate multiple mechanisms of brain vascular damage that contribute to ischemic injury independent of complement system activation.^3,12^ In addition to MBL-A’s greater ability to activate the complement system than MBL-C, a further explanation for MBL-A’s contribution to ischemic injury may be that it is activated earlier after ischemia than MBL-C. The gene encoding MBL-A (*Mbl1*) was overexpressed 24 h after ischemia, preceding the overexpression of the gene encoding MBL-C (*Mbl2*, 48 h). The temporally different overexpression could be regarded as a response to the earlier deposition of MBL-A in the brain vasculature (consumption)—6–12 h after ischemia—than that of MBL-C, which peaks later, at 24 h, according to our previous report.^2^ MBL isoforms are not directly synthesized by brain cells; thus, their presence in the brain depends on the deposition of circulating proteins on altered self-structures, such as the ischemic endothelium. It is likely that targeting MBL-A results in a stronger protective effect than targeting MBL-C owing to the role of MBL-A in the initial phases after ischemia, when it starts detrimental cascades that are hard to counteract at later stages. These cascades include—along with complement activation—inflammatory platelet activation,^12^ prothrombotic pathways,^11^ and immune cell activation.^20^ Whether the activation of specific cascades is linked to each MBL isoform remains to be clarified; however, the fact that depletion of both MBL isoforms results in the highest degree of protection supports the hypothesis that both isoforms play a role in ischemic brain injury, most likely activating different routes of damage. In other pathological settings, MBL isoforms may have a different behavior than that reported here for ischemia/reperfusion. Unlike after tMCAo, after traumatic brain injury, the presence of MBL-C in the brain is reported to occur more quickly after injury (30 min) than that of MBL-A.^14^ In line with this observation, MBL-C has been proposed as the main therapeutic target for traumatic brain injury.^15^ The different temporal patterns of MBL isoform deposition in different brain injury models indicate the availability of specific danger-associated molecular patterns for the response to brain insults.

Murine MBL isoforms were reported to differ in monosaccharide specificity, quite likely leading to preferential binding and potential accessibility to different microorganisms and altered self-structures.^16^ In brain ischemia, this effect may result in different abilities to bind the endothelial glycocalyx with postischemic modifications and possibly induce earlier MBL-A deposition than MBL-C deposition. On the basis of its carbohydrate specificity, MBL-C is the isoform most similar to human MBL, in agreement with genetic analysis, which reveals high homogeneity between *Mbl2* and the single human MBL gene.^16,21^ It has been proposed that MBL genes were duplicated prior to human-rodent divergence and that the human homolog of *Mbl1* was lost during evolution.^21^ However, all studies characterizing the similarities of the murine MBL isoforms with the human protein have been performed in vitro (complement activation assays and substrate affinity) or with samples from healthy mice. In a pathological setting such as brain ischemia, the functional similarities between the murine MBL isoforms and human MBL are underinvestigated. Although we suggest that MBL-A (less similar to human MBL) is the main contributor to brain ischemic injury, clinical data show that stroke patients with MBL deficiency develop less-severe injury than those without,^8^ mirroring the observations in the mouse knockout model. Human MBL is therefore a key contributor to postischemic damage. Accordingly, (1) human MBL may be used as early as mouse MBL-A after ischemia, since there is no competition for the substrate; (2) MBL may exert detrimental actions not requiring the activation of the complement cascade, such as those associated with coagulation system interactions, which are potentially important for stroke.

In conclusion, our study provides new insight into the specific importance of the murine MBL isoforms in brain ischemia/reperfusion injury. A few compounds that target MBL have been successful when systemically administered in preclinical models of acute brain injury,^2,15,22^ thus demonstrating that this pathway is druggable. Future preclinical works in rodents should target both isoforms to develop fully effective pharmacological tools. As an alternative, one could develop selective MBL isoform-targeting agents and define a treatment schedule based on the temporal pattern of each isoform’s presence/action. An additional approach could be to use newly developed humanized mice expressing human MBL under the control of the *Mbl1* promoter with deleted *Mbl* murine isoforms (MBL2 KI mice^23^). This model needs validation in an experimental setting such as ischemia, since, despite having a phenotype similar to that of WT mice, MBL2 KI mice have a baseline serum MBL2 expression level half of that of MBL-A in WT mice. Because it has a vascular site of action, MBL can be accessed easily, with no need to cross the blood–brain barrier, a critical limitation for other brain targets. The information provided here should be useful to identify and test selective molecules targeting MBL and possibly permit successful clinical translation of stroke research.

## Materials and methods

### Mice

Procedures involving animals and their care were conducted in conformity with institutional guidelines in compliance with national and international laws and policies. We used male C57BL/6 J mice 9–11 weeks old and weighing 26–28 g, either WT (the control strain as indicated in the strain datasheet for the mutated mice, visit: https://www.jax.org/strain/006122) or with targeted depletion of both (MBL^−/−^, purchased from Jackson Laboratories-USA and colonized at the Mario Negri Institute), or single MBL isoforms (MBL-A^−/−^ and MBL-C^−/−^, obtained at Mario Negri Institute by crossing MBL^−/−^ mice with WT mice and selecting appropriate colony founders). The protocols and details of this report are in accordance with the ARRIVE guidelines (http://www.nc3rs.org.uk/page.asp?id=1357, see the list provided as a supplementary file). We used only male mice since estrogens affect the ischemic outcome in experimental models.^24^ Examining the hormonal contribution to the ischemic lesion was beyond this work’s aim, which was to explore the specific roles of the MBL isoforms in brain ischemia.

### Focal cerebral ischemia

tMCAo was induced with a siliconized filament (7–0, Doccol Corporation) introduced into the right carotid artery and advanced to block the origin of the MCA for 60 min, as described previously.^2,25^ The surgery-associated mortality rate was 10%, with no difference among the genotypes.

### Behavioral deficits

At 48 h after tMCAo, mice were analyzed using a composite neurological score (range 0–56), as reported in Orsini et al.^2^

### Ischemic volume

Infarct volumes were calculated on 20 µm coronal brain cryosections stained with cresyl violet, integrating the infarcted areas after correction for the percentage of brain swelling owing to edema (determined by subtracting the area of the ipsilateral from that of the contralateral hemisphere).^2^

### Neuronal count

Three cresyl violet–stained 20-mm-thick coronal sections at −0.6, 0, and + 0.6 mm from the bregma were acquired from each mouse and visualized at ×20 magnification with an Olympus BX-61 Virtual Stage microscope, with a pixel size of 0.346 mm. Neuronal counting was performed by segmenting the cells in regions of interest designed in the ipsilateral cortex and striatum where the lesion was clearly detectable (pale cresyl violet staining, see Fig. 2a for the sampling strategy). The regions of interest on the contralateral side were positioned mirroring those on the ipsilateral side. A segmented circular signal smaller than the area threshold of 25 mm^2^, which is associated with glial cells,^15^ was excluded from the analysis. For quantification, we used Fiji software,^26^ and the neuronal cell number was expressed as the ratio of the number of neurons in the ipsilateral region of interest to that in the contralateral region of interest.

### Gene expression analysis

Livers were collected from anesthetized mice and immediately frozen on dry ice. Total RNA was extracted using an miRNeasy kit (Qiagen) according to the manufacturer’s instructions. Samples of total RNA (100 ng) were treated with DNase (Applied Biosystems, Foster City, CA, USA) and reverse transcribed with random hexamer primers using MultiScribe reverse transcriptase (TaqMan reverse transcription reagents, Applied Biosystems, Foster City, CA, USA). Primers were designed to span exon junctions to amplify only spliced RNA using PRIMER-3 software (http://frodo.wi.mit.edu/) and based on GenBank accession numbers (*Mbl1*: NM_010775.2; *Mbl2*: NM_010776.1, β-actin: NM_007393.3). The same starting concentrations of the cDNA template were used in all replicates. Real-time PCR was performed using Power SYBR Green according to the manufacturer’s instructions (Applied Biosystems). β-actin was used as the reference gene, and the relative gene expression levels were determined according to the ΔΔCt method (Applied Biosystems). Data are presented as the fold change compared with each control group. The primer sequences were as follows: *Mbl1* forward (fwd): cctgtcattggggaggatac and reverse (rev): atggaagcagaagcatggtc; *Mbl2* fwd: gaatccggggttaaaaggag and rev: tgatcgtagggctgcaattt; and β-actin fwd: cgcgagcacagcttcttt and rev: gcagcgatatcgtcatccat.

### Gene basal expression and microarray analysis

Basal expression levels of *Mbl1*, *Mbl2*, and *HSE-MSF* (C3) in *Mus musculus* were obtained from the online RNA-seq database available at http://www.brainrnaseq.org/ and originally published in ref. ^27^. Published microarray data were used to compare the gene expression levels (normalized log2 OD) in 3 h tMCAo and 24 h tMCAo mice with those in untreated WT mice (dataset number: GSE32529, published in ref. ^28^) using GEO2R software (NCBI).

### Sample collection for ELISAs

Blood was obtained from the vena cava of anesthetized mice. Clotting and complement activation were prevented by collecting samples in 10 mm ethylenediaminetetraacetic acid (EDTA). Plasma was centrifuged at 2000 × *g* for 15 min at 4 °C and stored at − 80 °C before analysis. Brain cortex homogenates were obtained after 3 min of perfusion with cold 0.01 m phosphate-buffered saline (PBS) to clear the blood. Each cortex sample was weighed and homogenized in 1% Triton X-100 lysis buffer (5 μl per 1 μg of weight) supplemented with protease (1 × PBS complete protease inhibitor cocktail, CPIC, Roche, USA) and phosphatase (1 μm 4-nitrophenyl phosphate, 4-NPP, Roche, USA) inhibitors.^29^ The homogenate was then centrifuged for 15 min at 10,000 rpm at 4 °C, and the supernatant was collected and stored at −80 °C until use. Extracted proteins were quantified with a NanoDrop ND-1000 spectrophotometer (Thermo Fisher).

### Plasma and brain protein levels

MBL isoforms were analyzed after sample incubation on mannan-coated (10 µg/mL) plates prepared according to a procedure reported previously.^18^ Plates were incubated for 1 h at 37 °C with EDTA-plasma diluted in barbital-buffered saline (BBS; 4 mm barbital, 145 mm NaCl, 2 mm CaCl_2_, 1 mm MgCl_2_ (pH 7.4)) to a final plasma concentration of 2.5% or with cortical homogenates (ipsilateral and contralateral to the lesion) diluted in BBS to a final concentration of 4 mg/mL total protein. Samples were then incubated for 1 h 30 min at room temperature (RT) with anti-MBL-A or anti-MBL-C antibodies (both 1 µg/mL, Hycult, # HM1035 and #HM1038, respectively). After washing, plates were incubated with an HRP-labeled goat anti-mouse IgG antibody (Santa Cruz, CA, USA), diluted to 0.4 µg/mL in wash buffer for 1 h 30 min at RT. After washing, the assay was developed by adding 100 µL of TMB substrate solution (TMB Substrate Kit, code 34021, Thermo Scientific, MA, USA; diluted 1:1 in H_2_O_2_ solution). The reaction was terminated by adding 100 μL of 2 m H_2_SO_4_, and the absorbance at 450 nm was measured using an Infinite M200 spectrofluorimeter managed by Magellan software (Tecan, CH).

### LP activity assay

We used serial plasma dilutions (10%, 5%, 2.5%, 1.25%, and 0.75% in BBS) and pooled plasma of mice belonging to the same experimental group, incubating the samples for 15 min on mannan- (10 µg/mL) or acBSA-coated (25 µg/mL) plates prepared according to a procedure reported previously.^18^ Plates were washed and incubated for 1 h 30 min at RT with a polyclonal anti-human-C3c antibody (Dako, A0062) diluted to 2.4 µg/mL in wash buffer. After washing, plates were incubated with an alkaline phosphatase-labeled goat anti-rabbit IgG antibody (Sigma A-3812) diluted to 1 µg/mL in wash buffer for 1 h 30 min at RT. After washing, the assay was developed by adding 100 µL of substrate solution (Sigma Fast p-Nitrophenyl Phosphate Tablets, Sigma). The absorbance at 405 nm was then measured using the Infinite M200 spectrofluorimeter managed by Magellan software (Tecan, CH).

### C3 deposition in the brain

Sections were stained using a polyclonal rabbit anti-C3 antibody (3.2 µg/mL, Novus Biologicals) followed by a secondary biotinylated antibody against rabbit IgG. Positive cells were stained with 3,3’-diaminobenzidine tetrahydrochloride (Vector Laboratories, CA, USA). For negative control staining, the primary antibody was omitted, and no staining was observed. Three 20 mm-thick coronal sections at −0.6, 0, and + 0.6 mm from the bregma were acquired for each mouse and visualized at × 20 magnification with the Olympus BX-61 Virtual Stage microscope, with a pixel size of 0.346 mm. The C3 signal was segmented over the ischemic ipsilateral cortex with Fiji software;^26^ the data are expressed as the percentages of stained area among the total area.^30^

### Experimental design and statistics

Mice were randomly allocated to surgery and assigned across cages and days. To minimize variability, all surgeries were performed by the same investigator. Subsequent behavioral, histological, immunohistological, and biochemical evaluations were performed by blinded investigators. Groups were compared by analysis of variance and post hoc testing as indicated in each figure legend. A parametric or nonparametric test was selected after the Kolmogorov–Smirnov test for normality to assess whether the data for the groups were normally distributed. The constancy of the variances was checked by the Bartlett test. Welch’s corrected one-way analysis of variance followed by the Games–Howell test was used for normally distributed data with unequal variances (Figs. 4b’ and 5a). The group size was defined pre hoc using the following formula: *n* = 2*σ*^2^f(*α*, *β*)/*Δ*^2^ (SD in groups = *σ*; type 1 error, *α* = 0.05; type II error, *β* = 0.2; percentage difference between groups, *Δ* = 30). For each measure, the standard deviation (SD) between groups was calculated on the basis of the composite neuroscore—set as the primary outcome—measured in WT mice in previous experiments using the same model (*σ* = 23, yielding *n* = 9.29).

To limit the use of animals, a post hoc power analysis test was performed for *n* = 6 on raw data for each experimental branch. The experiment was terminated at *n* = 6 for MBL-C^−/−^ mice, as it was unable to detect significant differences using a reasonable number of animals (*Δ* = 6.7, *σ* = 39.95; thus, the expected *n* = 567.28). A “health score” was obtained by stratifying the neuroscore, ischemic volume, and neuronal count data sets for WT mice into four groups according to quartiles. Each quartile was attributed a score ranging from 4 (good outcome) to 1 (bad outcome). The total score was the sum of the weighted scores of the three parameters, e.g., the neuroscore accounted for 50%, ischemic volume accounted for 25% and neuronal count accounted for 25% of the final score. The effect size (odds ratio) was calculated by a Chi-square test using the Woolf logit interval for computing the 95% confidence interval, stratifying mice in terms of good outcome (defined as a score = 4) vs. bad outcome (score < 4). Odds ratios are reported with a forest plot. Statistical analysis was performed with the standard software package GraphPad Prism (GraphPad Software Inc., San Diego, CA, USA, version 7.0); *p* values lower than 0.05 were considered significant.

## Data Availability

The data sets generated and/or analyzed during the current study are available in the Figshare repository, 10.6084/m9.figshare.7378400.
